# Nucleotide‐Binding Oligomerization Domain 2 in Signaling, Immunity, and Mycobacterial Infection

**DOI:** 10.1002/iid3.70272

**Published:** 2025-09-28

**Authors:** Yi Wang, Zihao Mi, Hong Liu, Furen Zhang

**Affiliations:** ^1^ Dermatology Hospital of Shandong First Medical University Jinan China; ^2^ Shandong Provincial Institute of Dermatology and Venereology, Shandong Academy of Medical Sciences Jinan China

**Keywords:** Crohn's disease, immune homeostasis, mycobacterial infection, NOD2, signaling pathways

## Abstract

**Introduction:**

Nucleotide‐binding oligomerization domain 2 (NOD2) functions primarily as a cytoplasmic pattern recognition receptor (PRR) that detects muramyl dipeptide (MDP), a conserved bacterial cell wall component, thereby playing a pivotal role in pathogen surveillance. However, emerging evidence reveals that NOD2 exerts broad immunomodulatory effects beyond its canonical role as a PRR, though its effects can be contradictory, depending on genetic background, immunological microenvironment, and disease context. In this review, we integrate recent advances in understanding NOD2's functional plasticity and provide novel insights into its regulatory mechanisms in immune responses and mycobacterial infections.

**Methods:**

A literature search was conducted using the PubMed database with keywords including NOD2, signaling pathways, immune homeostasis, autophagy, trained immunity, and mycobacterial infection. Relevant information was extracted from the retrieved research articles and reviews and summarized.

**Results:**

We delineated the MDP‐dependent and ‐independent mechanisms of NOD2 activation and their downstream signaling cascades. We also elaborated on the classical immune responses orchestrated by NOD2, and its remarkably multifaceted noncanonical roles in regulating immune homeostasis by modulating autophagy, acting synergistically with toll‐like receptor pathways to fine‐tune inflammation, and balancing trained immunity and immune tolerance. Furthermore, we examined the multifaceted immunoregulatory functions of NOD2 in host defense against mycobacterial infections.

**Conclusions:**

We propose that further research is required to clarify the various roles of NOD2 across diverse genetic backgrounds, microenvironmental contexts, and disease paradigms. Such studies will provide critical mechanistic insights to inform the development of precision‐based therapeutic strategies targeting NOD2.

## Graphical abstract

NOD2 is a cytoplasmic receptor that detects bacterial components and plays key roles in immune surveillance, but it also has diverse noncanonical immunomodulatory functions beyond pathogen recognition. This review explores NOD2's activation mechanisms, classical and nonclassical immune roles, and its impact on mycobacterial defense, highlighting the need for further research to guide targeted therapies. Understanding NOD2's varied functions across genetic and disease contexts could advance precision medicine approaches.

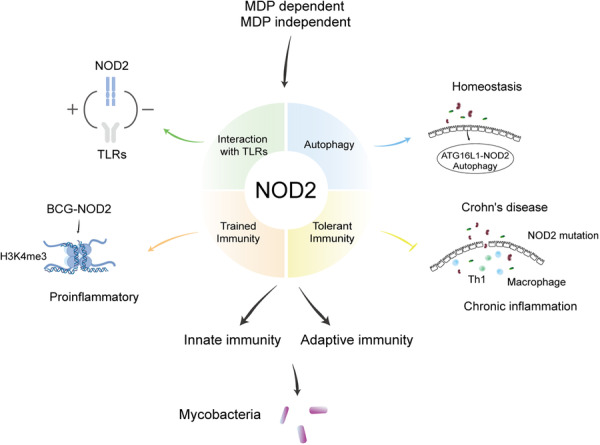

## 1 Introduction

The innate immune system acts as the first line of defense against bacterial infections, and its dysfunction results in the development of infectious, inflammatory, and autoimmune diseases. Pattern recognition receptors (PRRs) are expressed outside or inside innate immune cells, and recognize the pathogen‐associated molecular patterns (PAMPs) conserved in different pathogens, triggering specific signaling pathways that regulate the early host responses. Currently known types of PRRs include the toll‐like receptors (TLRs) and C‐type lectin receptors located on the cell membrane, and the NOD‐like receptors, RIG‐I‐like receptors, and AIM2‐like receptors expressed in the cytoplasm [1, 2]. Nucleotide‐binding oligomerization domain 2 (NOD2) is an intracellular PRR that recognizes muramyl dipeptide (MDP), the conserved minimal bioactive peptidoglycan (PGN) motif present in bacterial cell walls, and activates various signaling pathways, thereby driving pro‐inflammatory and antipathogenic responses [3].

Crohn's disease (CD) is a chronic, recurrent, inflammatory bowel disease that manifests as abdominal pain, diarrhea, and intestinal obstruction, and is mainly caused by the disruption of normal intestinal homeostasis. Extensive studies have shown that NOD2 mutation is one of the main causes of CD [4, 5]. In individuals with NOD2 deficiencies, the ability to recognize and kill bacteria is weakened, leading to the destruction of the intestinal mucosal barrier. Consequently, microorganisms translocate into the lamina propria of the intestinal mucosa. Immune cells respond to the exposed microorganisms by continuously secreting cytokines, leading to the development of chronic intestinal inflammation [6, 7]. NOD2 plays a dual role: it not only activates inflammation to protect the integrity of the intestinal epithelial barrier during acute enteritis, but also acts as a crucial negative regulator in chronic enteritis, combating excessive inflammation and mitigating tissue damage [8, 9, 10, 11]. Thus, NOD2 plays a pivotal role in maintaining intestinal immune homeostasis. Furthermore, recent discoveries have demonstrated its involvement in autophagy, its collaboration with TLRs, and its crucial function in balancing immune tolerance and trained immunity.

In addition to its role in inflammatory diseases, NOD2 also plays a significant part in the host's immune defense against invading pathogens [12], particularly mycobacteria. Immunological, genetic, and chemical studies have demonstrated a correlation between NOD2 and mycobacterial infection [13]. The polymorphism of the *NOD2* gene is related to the risk of tuberculosis (TB) [14], an infectious disease caused by *Mycobacterium tuberculosis* (*Mtb*), which poses serious ongoing challenges to global public health. In 2022, approximately 10.6 million people worldwide suffered from TB, making it the second most deadly infectious pathogen after COVID‐19 [15]. Although the Bacillus Calmette‐Guérin (BCG) vaccine offers protection against severe childhood TB, its effectiveness against pulmonary TB in adults is limited, underscoring the urgent need for improved TB vaccines [16]. It is noteworthy that the immune training mechanism induced by BCG vaccination is intimately linked to NOD2. Furthermore, a genome‐wide association study in 2009 showed that *NOD2* is a susceptibility gene for leprosy, which is caused by infection with *Mycobacterium leprae* or *Mycobacterium lepromatosis* [17]. Leprosy is primarily characterized by skin lesions and peripheral nerve damage, with the potential development of permanent deformities or disabilities in its later stages [18]. Notably, mycobacteria produce N‐glycolyl MDP, a specific fundamental molecule with stronger immunostimulatory activity toward NOD2 than MDP [19, 20, 21]. Although these clues are thought‐provoking, accumulating research has shown that NOD2 has multifaceted functionality, with sometimes contradictory roles that depend upon the genetic background, the immunological microenvironment, and the disease context. In this review, we integrate recent advances in understanding the functional plasticity of NOD2, and provide novel insights into the regulatory mechanisms underlying its roles in both immune responses and mycobacterial infections.

## 2 NOD2 Signaling

### 2.1 Functions of NOD2 Domains

NOD2, encoded by the *CARD15* gene, consists of 1040 amino acids, with a molecular weight of 110 kDa. NOD2 is expressed in various types of cells, including macrophages, dendritic cells (DCs), and epithelial cells [22]. It is the sensor of MDP, a PAMP that is ubiquitously present on Gram‐negative and ‐positive bacteria [3, 23]. Iyer et al. suggested that polymeric PGN is a better pro‐inflammatory inducer of NOD2‐dependent responses than MDP, although the possibility of contaminants in the polymeric PGN cannot be completely excluded [24].

**Figure 1:**
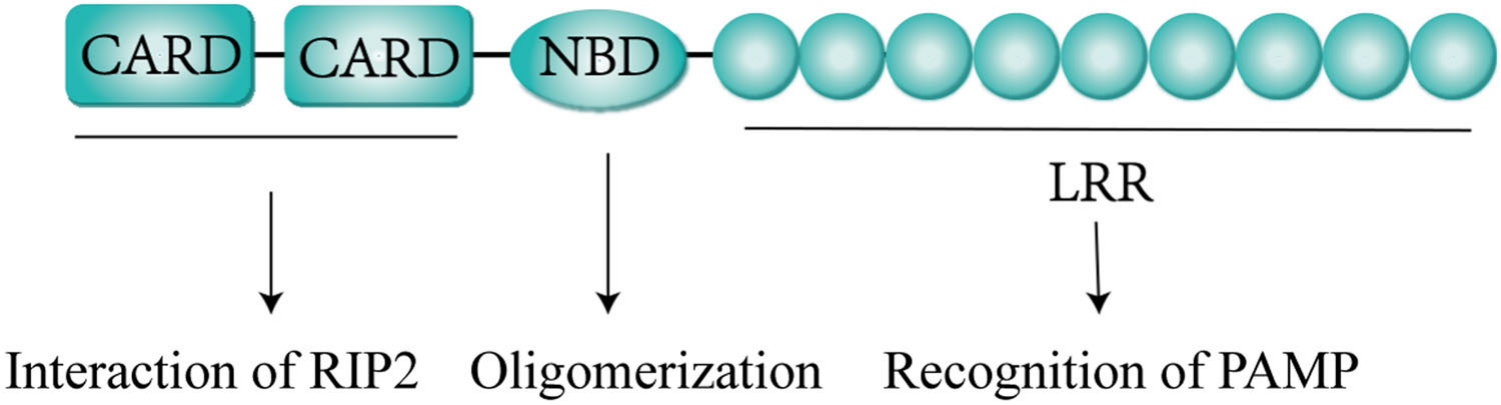
Three domains of NOD2. CARDs interact with RIP2. NBD is responsible for the oligomerization of NOD. LRR recognizes MDP.

The structure of the NOD2 protein contains three domains: a leucine‐rich repeat (LRR) domain at the carboxy terminal, a nucleotide‐binding domain (NBD) in the middle, and two caspase recruitment domains (CARDs) at the amino‐terminal [25, 26] (Figure 1). The LRR domain of NOD2 is involved in the recognition of MDP, which triggers conformational changes in the protein [27], thereby contributing to the NBD‐mediated oligomerization of NOD proteins [28]. Individuals with NOD2 variations located in the LRR region (Arg702Trp, Gly908Arg, or Leu1007fsinsC) are susceptible to CD [29, 30]. However, mutations Arg334Trp, Arg334Gln, and Leu469Phe in the NBD region lead to defective NOD2 oligomerization and cause Blau syndrome [31]. The CARDs of NOD2 interact with the CARD of receptor‐interacting protein 2 (RIP2), as has been demonstrated at the structural level [32, 33]. The structural domain architecture of NOD2 underpins its versatile signaling capacities, and in subsequent sections, we discuss the mechanistic underpinnings by which this PRR activates downstream signaling networks via both MDP‐dependent and ‐independent pathways.

### 2.2 MDP‐Dependent Activation of NOD2

The activation of NOD2 signaling pathways can be divided into two categories: MDP‐dependent and MDP‐independent. The MDP‐dependent pathways have been extensively investigated. Cells phagocytose bacteria and form phagosomes, which fuses with lysosomes and then further mature into phagolysosomes, in which the bacteria are digested into PGN, the major component of the bacterial cell wall [34, 35]. The basic unit of PGN, MDP, must be phosphorylated by N‐acetylglucosamine kinase before it is recognized by NOD2 [36]. In macrophages and DCs, the SLC15 family endosomal peptide transporter protein 1 PHT1 (SLC15A4) facilitates the transmembrane transport of MDP, recruiting NOD2. Endosomes act as signaling platforms for triggering the innate immune response [3, 37, 38, 39]. Acidic chemicals that disrupt the endosome inhibit the NOD2‐induced activation of NF‐κB, which may be due to the dependence of ligand transport on proton gradients as the energy source [40]. HPEPT1 located on the plasma membrane transports MDP directly into the cytosol [41, 42]. Heat shock protein 70 is a positive regulator of NOD2 signaling, prolonging the half‐life of NOD2 and enhancing NF‐κB activity [43].

Although NOD2 was initially described as a cytoplasmic receptor, it has been shown that the membrane targeting of NOD2 is required for NF‐κB activation [22]. This is illustrated by the CD‐related NOD2 loss‐of‐function mutation 3020insC, which, although fully capable of binding MDP, fails to bind to the membrane and therefore cannot detect bacterial invasion [4, 44]. Although NOD2 is anchored to membranes by cytoskeletal components (vimentin) and binding proteins (FRMPD2, FERM, and PDZ structural domain 2), or to endosomes by endosomal proteins (SLC15A) [38, 45, 46], studies have shown that the palmitoylation of NOD1/2 oligomers is required for both the steady‐state binding of NOD2 to the membrane and MDP‐induced signaling pathways. During this process, the presence of zinc‐finger and aspartate‐histidine‐histidine‐cysteine 5 (ZDHHC5) and its enzymatic activity are essential for the proper recruitment of NOD1/2 to the site of bacterial entry and the formation of phagosomes [47]. However, how ZDHHC5 is attracted to these bacterial entry sites is still unknown [47].

### 2.3 MDP‐Independent Activation of NOD2

**Figure 2:**
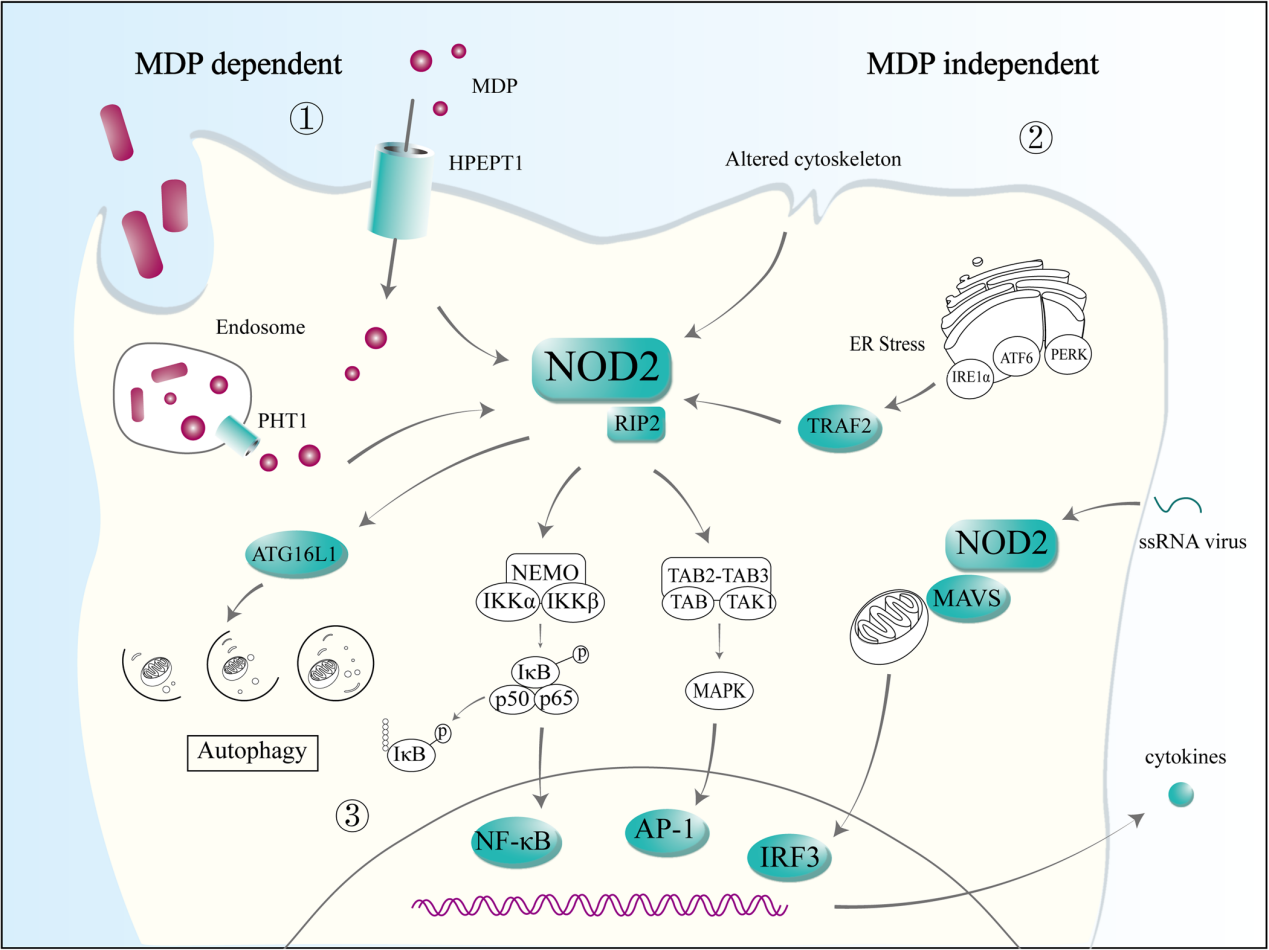
Activation and signaling pathways of NOD2. (1) MDP‐dependent: NOD2 detects MDP from inside and outside the bacteria. PHT1, an endosomal peptide transporter protein of the SLC15 family, is transported into the cytosol by host cells following the phagocytosis of bacteria and subsequent bacterial degradation. The plasma membrane protein HPEPT1 directly transports peptidoglycan into the cytoplasm. (2) MDP‐independent: the involvement of NOD2 in viral ssRNA recognition, followed by its interaction with MAVS, induces IRF3 activation and triggers *IFNB* gene expression. ER stress recruits TRAF2 and facilitates NOD2 activation in response to unfolded proteins. Alterations in the cytoskeleton can also stimulate NOD2. (3) NOD2 activation followed by CARD–CARD oligomerization induces RIP2 activation and activates NF‐κB and MAPKs, which trigger the expression of inflammatory cytokines. NOD2‐mediated autophagy is essential for the control of bacterial.

In contrast to its MDP‐dependent activation, the mechanism of MDP‐independent activation of NOD2 is ambiguous [48]. It has been shown that viruses, such as respiratory syncytial virus and Middle East respiratory syndrome coronavirus, can activate NOD2 [49, 50]. Once viral single‐stranded RNA (ssRNA) is recognized, NOD2 enhances interferon‐β (IFN‐β) expression through the activation of interferon regulatory factor 3 (IRF3) by mitochondrial antiviral signaling protein (MAVS) [49]. Keestra‐Gounder et al. also proposed a link between endoplasmic reticulum (ER) stress and the increase in interleukin‐6 (IL‐6) secretion via NOD1/NOD2 signaling, independently of MDP [51]. The accumulation of unfolded or misfolded proteins, as well as viral or bacterial infections, can induce the unfolded protein response (UPR) in the ER, which is a protective response that limits the cellular damage caused by ER stress [52]. The UPR involves three transmembrane receptors, protein kinase RNA‐like endoplasmic reticulum kinase (PERK), activating transcription factor 6 (ATF6), and inositol‐requiring enzyme 1 (IRE1α). In mice, the intraperitoneal injection of *Bifidobacterium abortus* triggered its type IV secretion system, which secreted the bacterial effector protein VceC, which induced ER stress. IRE1α recruited TNF receptor associated factor 2 (TRAF2) to the ER membrane and induced NOD1/NOD2 activation to promote IL‐6 production via the NF‐κB pathway [51, 53] (Figure 2).

The cytoskeletal protein vimentin performs cellular functions by interacting with the LRR structural domain of NOD2, and recruiting NOD2 into the cell membrane, affecting NF‐κB activation, autophagy, and bacterial processing [54]. The interaction of the cytoskeletal regulator Rac family small GTPase 1 (RAC1) and Rho guanine nucleotide exchange factor 7 (ARHGEF7; also known as PAK3BP) negatively regulates NOD2 activation, and the downregulated expression of both proteins enhances MDP‐driven NOD2 activation [55, 56]. Consistent with this, in bone‐marrow monocytes and intestinal epithelial cells, the disruption of the actin cytoskeleton by cytochrome D also increases NOD1‐ and NOD2‐mediated NF‐κB activation, even in the absence of PGN, suggesting that cytoskeletal alterations can trigger the activation of NOD2 [56].

### 2.4 Signaling Pathways Downstream From NOD2 Activation

The activation of NOD2 predominantly initiates downstream gene expression through two signaling pathways, the NF‐κB and mitogen activated kinase‐like protein (MAPK) pathways (Figure 2). In response to ligand binding, the RIP2 recruited by NOD2 is extensively and selectively ubiquitinated, when methionine 1‐linked (M1) ubiquitin binds the IκB kinase (IKK) complex (IKK‐β, IKK‐α, and NEMO). The E3 ubiquitin ligase X‐linked inhibitor of apoptosis protein (XIAP), cellular inhibitor of apoptosis protein 1 (CIAP1), CIAP2, TRAF2, TRAF5, and the linear ubiquitin assembly complex (LUBAC) are also involved in this process [57, 58, 59, 60]. The RING domain of XIAP recruits LUBAC (a trimeric complex that contributes to the modification of linear ubiquitination) to RIP2, which is essential for the NOD2‐dependent response [60]. Consistent with this observation, mutations Leu207Pro and Val198Met in the BIR2 domain of XIAP lead to the inactivation of ubiquitin (Ub) ligase and impair NOD2‐dependent immune signaling [61]. Several deubiquitinating enzymes also negatively regulate the activation of NOD2, including CYLD lysine 63 deubiquitinase (CYLD), A20, and OTU deubiquitinase with linear linkage specificity (OTULIN) [62, 63]. Interferon regulatory factor 4 (IRF4) acts as a negative regulator of NOD2 by inhibiting RIP2 ubiquitination in human DCs, thereby reducing NOD2‐dependent NF‐κB activation [64]. The activated IKK complex acts as an IκB kinase to phosphorylate the IκB protein, followed by its auto‐ubiquitination and proteasomal degradation, releasing the NF‐κB complex, which is translocated into the nucleus to promote cytokine expression [65].

In parallel, NOD2 signaling via lysine‐63‐linked ubiquitin initiates the recruitment of the TAK1–TAB2–TAB3 complex [66], allowing the activation of extracellular signal‐regulated kinase 1 (ERK1), ERK2, JUN N‐terminal kinase (JNK), and p38, which regulate the transcription factor AP‐1 through the phosphorylation of MAPK [67]. The transcription factors NF‐κB and AP‐1 alone or in combination induce cytokine expression in the nucleus [44, 68, 69]. The activation of NF‐κB and MAPK downstream from NOD2 not only drives the secretion of inflammatory cytokines but also influences immune homeostasis by regulating the expression of autophagy‐related 16‐like 1 (ATG16L1) and epigenetic modifications, which provides a molecular basis for the multifunctionality of NOD2 in immunity.

## 3 Multifaceted Roles of NOD2 in Immunity

### 3.1 Innate Immunity

Innate immunity is a host response that exerts a direct or nonspecific effect on pathogens by activating immune cells and inflammatory mediators. NOD2 drives the inflammatory response to bacteria through the activation of the NF‐κB and MAPK pathways, both of which trigger increases in the expression of pro‐inflammatory factors, including IL‐1β, tumor necrosis factor α (TNF‐α), IL‐6, IL‐8, IL‐12p40, and chemokines CCL2 and CXCL2 [70, 71, 72, 73]. This also occurs in neutrophils [74, 75, 76].

In addition to the activation of each of the PRRs, the multiple signaling pathways of NOD1, NOD2, and TLR act synergistically in stimulating intracellular signaling cascades to produce more pro‐inflammatory factors, which are involved in the early innate immune response to infection. Stimulation of mouse‐derived peritoneal macrophages or THP‐1 by the coupling of a TLR and a NOD‐like receptor agonist not only enhances downstream NF‐κB activation and the release of pro‐inflammatory factors, but also accelerates bacterial clearance from the circulation and internal organs through enhanced phagocytosis by macrophages, which protects the mice from microbial sepsis‐induced lethality [77, 78].

The excessive production of pro‐inflammatory cytokines causes physiological and immunopathological damage to tissues, so the macrophage tolerance induced by MDP, lipopolysaccharide (LPS), or other bacterial ligands is considered a protective mechanism [79]. Consistent with this observation, there is a lack of cross‐tolerance between TLRs and NOD2 signaling, and macrophages are heavily dependent on NOD1 and NOD2 when TLR signaling is tolerance inhibited [80]. Compared with wild‐type mice, Nod1^−/−^Nod2^−/−^ and RIPK2^−/−^ mice showed significantly impaired survival and bacterial clearance after exposure to LPS or *Escherichia coli* in vivo, with reduced expression of the promotional cytokine IL‐6 [80]. This delicate balance between immune activation and tolerance highlights the unique position of NOD2 as a molecular switch that can either amplify or restrain the inflammatory response, depending upon the immunological context.

### 3.2 Adaptive Immunity

Once pathogenic microorganisms have breached the various barriers of the innate immunity, adaptive immunity, which is heavily dependent on DCs, is required to activate antigen‐specific T and B lymphocytes. The failure of NOD2 to drive Th2‐regulatory signaling leads to intestinal mucosal disorders, which is one pathway toward the development of CD [81, 82]. MDP induces the Th2‐cell‐dependent secretion of IL‐4 and IL‐5, which requires the upregulation of nonhematopoietic thymic stromal lymphopoietin protein (TSLP) and the expression of OX40 ligand (OX40L) on DCs. The TSLP–OX40L axis is essential for DCs to act as antigen‐presenting cells (APCs) to regulate the induction and functional alterations of Th2 cells [82]. NOD2 also enhances IL‐23 secretion in DCs, thereby promoting the differentiation of CD4^+^ T cells to TH17 cells and the secretion of IL‐17. This may be responsible for bacterial clearance, as demonstrated in NOD2‐mutant DCs from some CD patients [83].

The coordination of NOD2 with other signaling pathways also plays an important role in influencing NOD2‐mediated adaptive immunity. Under combined treatment with monophospholipid lipid A (MPLA, a TLR4 agonist) and MDP (a NOD2 agonist), more‐intense cellular and humoral immune responses were observed in bone‐marrow‐derived cells (BMDCs), such as the higher expression of the maturation/activation markers MHCII, CD80, and CD86, the proliferation of splenic CD4^+^ and CD8^+^ T cells, and the elevation of IFN‐γ and immunoglobulin G (IgG), compared with the use of either single stimulant [77]. TLR2 acts synergistically with NOD2 to signal through TANK binding kinase 1 (TBK1) to PI31, which stabilizes the immunoproteasome by interacting with SEC16 homolog A, endoplasmic reticulum export factor (SEC16A) on the ER, promoting the presentation of major histocompatibility complex I (MHCI) antigen on DCs and initiating the CD8^+^ T‐cell response [84]. Similarly, the costimulation of NOD2 with the TLR2 ligand also promotes the production of the Th1‐polarized cytokine IL‐12 by macrophages, which initiates antigen‐specific T‐ and B‐cell immunity in vivo [81]. In a recent study, a synthesized dual NOD2/TLR7 agonist adjuvant displayed strong Th1‐biased adjuvant activity upon the activation of peripheral blood mononuclear cells (PBMCs) and BMDCs, which could not be compared with that of a single receptor agonist [85]. The chemical coupling of PRR agonists provides strong evidence of cross‐talk in signaling pathways and may also facilitate the development of novel vaccines in the future.

### 3.3 Trained Immunity and Tolerant Immunity

In recent years, there has been a growing realization that immune memory is not an exclusive characteristic of adaptive immunity. Innate immune cells appear capable of acquiring memory characteristics after a transient stimulus, resulting in a more powerful response when challenged twice, which is known as “trained immunity.” Trained immunity explains some of the heterologous effects of vaccines, in that it increases protection against secondary infections [86]. Mouse‐ or human‐derived cells prestimulated with BCG displayed NOD2‐induced trained immunity, showing nonspecific protection against infection and increased pro‐inflammatory cytokines IFN‐γ, TNF‐α, and IL‐1β [87]. Mice with severe combined immune‐deficiency, which lack T and B cells, were injected with lethal *Candida albicans* 2 weeks after the injection of BCG or MDP. Survival was significantly higher in the BCG‐vaccinated mice than in saline‐injected control mice [87]. Similarly, the activation of NOD2 by MDP in macrophages/monocytes drives strong innate immune memory and an adaptive response that can strengthen antiviral defenses, partly preventing SARS‐CoV2 epidemics [88]. These studies demonstrated that NOD2 plays a role in the induction of innate trained immunity.

As well as its involvement in trained immunity, NOD2 is involved in the state of hyporesponsiveness to specific antigens, called “immune tolerance.” This is a negative feedback regulatory mechanism that limits the health costs from excessive immune damage and prevents immunopathology [89]. NOD2‐induced immune tolerance is relevant to CD, which is characterized by a dysregulation of immune homeostasis due to excessive inflammation of the intestinal flora [90]. Compared with macrophages derived from normal volunteers, a higher TNF‐α, IL‐8, IL‐12, and IL‐1β levels were observed in macrophages from volunteers at risk of CD harboring the Leu1007insC NOD2 variant [91]. Studies have shown that the MDP‐mediated expression of IRF4 and ATG16L1 negatively regulates the TLR2‐RIP2‐NF‐κB and MAPK signaling pathways, by inhibiting the expression of ubiquitination ligases CIAP1 and CIAP2 of RIP2, leading to the reduced production of pro‐inflammatory cytokines [92, 93, 94]. This is why the preinjection of MDP in mice can prevent the development of colitis [93, 95]. However, studies have shown that MDP‐induced NOD2 signaling can also enhance the production of IL‐8, TNF, and IL‐1β through TLR pathways [96]. Researchers believe that NOD2 influences TLR‐mediated cytokine responses in either a negative or positive manner, depending upon the context, allowing or even promoting the development of host defense elements before exerting its primary tolerogenic effects. These different outcomes may be related to the dosage and/or mode of administration [93]. Furthermore, the IRF4‐dependent tolerance activated by MDP is also linked to the metabolism, alleviating adipose inflammation and insulin resistance in obese mice [97]. In addition to TLR2, recent studies have shown that NOD2 inhibits TLR9‐induced IFN‐α production in monocytes and DCs through a mechanism dependent upon deubiquitinating enzyme A, thus protecting mice from dextran‐sulfate‐sodium‐induced colitis [98].

As well as the collaboration of NOD2 with TLRs, it is also proposed that the immune tolerance caused by chronic stimulation with MDP is due to the destabilization of NOD2 or its signaling pathways. Following the action of MDP, the rapid dissociation of the heat shock protein 90 (HSP90) complex from NOD2 and the binding of the suppressor of cytokine signaling 3 (SOCS3) protein to NOD2 accelerate the process of NOD2 degradation, resulting in a weakened host response when confronted with either MDP or *Citrobacter rodentium* [99, 100]. Consistent with this, the degradation of RIP2 mediated by transmembrane protein zinc and ring finger 4 (ZNRF4) is a negative regulatory mechanism of the NOD2‐induced NF‐κB, cytokine, and antimicrobial responses. MDP‐tolerant mice lacking ZNRF4 displayed enhanced control of *Lactobacillus monocytogenes* infection [101]. NOD2 tolerance may also be caused by powerful negative feedback. Acute NOD2 stimulation promotes inflammation by inducing the activation of interleukin 1 receptor associated kinase 1 (IRAK1), whereas chronic NOD2 stimulation inhibits IRAK1 activity, induces the expression of the IRAK1 inhibitory protein IRAK‐M, and significantly reduces cytokines TNF‐α, IL‐8, and IL‐1, which promotes tolerance of the luminal flora and maintains the balance of intestinal immune tolerance [91]. The NOD2‐mediated early secretion of IL‐1Rα, IL‐10, and TGF‐β is important for their decline during prolonged NOD2 stimulation, and the inhibition of the NOD2 pathway and the selective blockade of the autocrine secretion of these cytokines reverses NOD2 tolerance [102].

A model of *Borrelia burgdorferi* disease has also been proposed, in which NOD2 plays a potentiating role in activating inflammation during early infection, but reduces tissue damage by inducing tolerance after prolonged exposure to the organism [89]. Although the inflammatory response of tolerant macrophages to bacterial infection was relatively weakened, this is partially offset by the accelerated maturation of NF‐κB‐dependent phagosomes, enhanced bactericidal activity, and the upregulated expression of lysosomal enzymes and membrane transport regulators (Rab10 and Acp5) [103].

The multifunctionality of NOD2 in both innate and adaptive immune signaling pathways allows it to induce trained immunity or tolerant immunity through distinct mechanisms. This functional dichotomy positions NOD2 as a promising therapeutic target for inflammatory and infectious diseases. However, the direction of NOD2‐mediated immune regulation is influenced by the genetic background and the immune microenvironment, so comprehensive and in‐depth studies are required to clarify its intricate regulatory mechanisms and to establish a theoretical foundation for developing therapeutic strategies.

### 3.4 NOD2 and Autophagy

In addition to regulating the classical innate and adaptive immune processes, NOD2 also contributes to immune homeostasis by participating in the modulation of autophagy. Autophagy is a highly conserved process in eukaryotic cells, maintaining homeostasis by degrading cellular proteins and organelles. NOD2 and the autophagy protein ATG16L1 are two of the most important proteins associated with CD. Several studies have confirmed that ATG16L1 plays important roles in the NOD2 pathway, including in pathogen targeting, the induction of autophagy, antigen presentation, and cytokine production. ATG16Ll promotes the local conversion of LC3‐I to LC3‐II by binding to ATG5–ATG12, and is recruited to the plasma membrane at bacterial entry sites by NOD2. The direct binding of NOD2 to ATG16L1 leads to the rapid formation of autophagosomes around invading bacteria [104]. The NOD2 ligand MDP induces the formation of autophagosomes in human DCs, thereby enhancing the clearance of bacteria and MHC‐II‐associated antigen presentation, which requires the involvement of the autophagy proteins Atg5, Atg7, Atg16L1, and RIP2 [105]. NOD2 also promotes the expression of cytokines IL‐1β, IL‐6, IL‐8, and TNF‐α through ATG16L1‐associated autophagy [106, 107, 108]. The number of intracellular *E. coli* was increased in *Atg16L1* ^−/−^ and *Nod2* ^−/−^ mice in vitro and in vivo models [107, 109].

However, ATG16L1 also interacts with IKK, an important link in the NOD2 pathway that activates the transcription factor NF‐κB, to protect cells from excessive anti‐inflammatory effects. IKK is involved in stabilizing ATG16L1 at Ser278, inhibiting ER stress, and secreting cytoprotective IL‐18, which may be involved in counteracting the pro‐inflammatory effects triggered by classical IKK/NF‐κB activation [110]. NOD2 also clears excess reactive oxygen species (ROS) through ATG16L1‐mediated mitochondrial autophagy, exerting a cytoprotective effect [111]. The mechanisms identified in these studies highlight the dual role of NOD2 in maintaining immune homeostasis through the modulation of autophagy, further emphasizing its sophisticated role in balancing organismal homeostasis.

## 4 NOD2 and Mycobacterial Infection

### 4.1 Association Between NOD2 Variants and Susceptibility to Mycobacterial Infection

The most well‐known association between NOD2 variants and mycobacterial infection was reported in 2009 in a genome‐wide association study of leprosy [17], which was later been successively validated in Vietnam, India, and ethnically mixed Amazonian populations [112, 113, 114]. Subsequent immunological experiments confirmed that human NOD2 recognizes the structurally unique MDP of *M. leprae* [115]. Unlike other mycobacteria, *M. leprae* produces an N‐glycolyl MDP structure, which is more readily recognized by NOD2 than MDP [20]. Unlike TLR2/1 ligands, MDP acting on NOD2‐activated monocytes is more likely to induce the rapid differentiation of monocytes into CD1^+^ DCs and to induce MHC‐I‐associated antigen presentation by an IL‐32‐dependent mechanism, which was also confirmed in leprosy lesions [116]. Although the association between NOD2 variants and TB has also been widely investigated, the results of these studies have not reached very consistent conclusions (unlike those for leprosy), which may be related to the high heterogeneity among different *Mtb* strains [14]. Nontuberculous mycobacteria (NTM) constitute another significant category of mycobacterial species, but research into the link between NOD2 variants and NTM infections has been limited. Notably, recent studies have identified an association between specific NOD2 mutations (p.Glu778Lys and p.Gly908Arg) and recurrent infections caused by *M. abscessus* [117]. The association between NOD2 variants and mycobacterial infections provides reliable genetic evidence for investigating the role of NOD2‐related immune processes in the pathogenesis of mycobacterial infections.

### 4.2 NOD2‐Mediated Innate and Adaptive Immunity Against Mycobacterial Infection

Because mycobacteria are a typical class of intracellular pathogenic bacteria, the role of NOD2—a cytosolic PRR—in mycobacterial infections has long been a focal point for immunologists. Moreover, the unique molecular structure of mycobacterial MDP induces stronger immune responses than those of other pathogens, underscoring the critical role of NOD2 in mycobacterial infections. Unlike other bacteria, mycobacteria produce a specific enzyme called N‐acetyl muramic acid hydroxylase (NamH), which converts the basic unit of the mycobacterial cell wall, N‐acetyl MDP, to N‐glycolyl MDP, with stronger stimulatory activity [19, 21]. In *Mtb*‐infected monocyte‐derived macrophages (MDMs), *NOD2* transcripts are upregulated up to 10‐fold, *NOD2* mRNA up to 2–3‐fold, and NOD2 protein up to 10‐fold [118, 119].

In human‐ and mouse‐derived cells infected with mycobacteria, NOD2 produces a large number of immune effectors through the innate immune response, including TNF, IL‐1β, IL‐6, IL‐12, IL‐32, type I IFNs, and nitric oxide (NO). TNF‐ and IL‐1R‐deficient mice fail to control mycobacteria, and with an increased bacterial burden, BCG exacerbated lung inflammation, reduced IL‐23, and was associated with rapid death [120, 121, 122, 123]. *Mtb*‐infected bone‐marrow–derived macrophages activate IRF5 and induce IFN‐α/β transcription via the NOD2‐RIP2‐TBK1 signaling pathway, which induces a bactericidal effect [124]. However, the capacity of NOD2‐mediated type I IFNs to clear mycobacteria is controversial. It has been suggested that type I IFNs encourage the cell‐to‐cell spread of mycobacteria by secreting CXCL chemokines and inducing necrotic apoptosis, but it is not yet known how type I IFNs lead to necroptosis [125, 126]. However, some in vivo and in vitro experiments in mice suggested that type I IFNs enhance the expression of inducible nitric oxide synthase (iNOS) and eliminate mycobacteria through NO production [127, 128]. Genetic disorders affecting the immune function of type I IFN underlie severe mycobacterial infections [129, 130, 131]. Therefore, whether IFN is beneficial or detrimental under specific circumstances must be investigated in future studies.

DCs are a bridge between the innate and adaptive immunity. Signaling through NOD2 and TLR4 (N_2_T_4_) enhances the efficacy of DCs and the potency to kill *Mtb* in vitro and in vivo, mainly via the following mechanisms: (1) the secretion of effectors IL‐6, IL‐12, IFN‐γ, TNF‐α, and NO; (2) the induction of autophagy; (3) an increase in CCR7, which is known to improve the migration of immune cells to the lymph nodes; and (4) an increase in the number of effector memory CD4^+^ and CD8^+^ T cells. N_2_T_4_ also lowers the dose of isoniazid required to treat TB (one‐fifth of the recommended concentration), and significantly reduces in lung bacterial load [132, 133, 134]. The role of NOD2 in adaptive immunity was also confirmed by in vivo experiments in mice, insofar as *Nod2* ^−/−^ mice infected with *Mtb* and treated with BCG produced fewer Th1‐type cytokines and showed reduced recruitment of CD8^+^ and CD4^+^ T cells in the later phase of the infection than wild‐type mice, and had a higher mortality rate [135]. N_2_T_4_ combination therapy activates the bridging function of DCs between innate immunity and adaptive immunity, which not only enhances the multilevel killing of *Mtb* but also significantly reduces the dose of antibiotic required, offering a breakthrough solution for drug‐resistant TB and latent infections.

**Figure 3:**
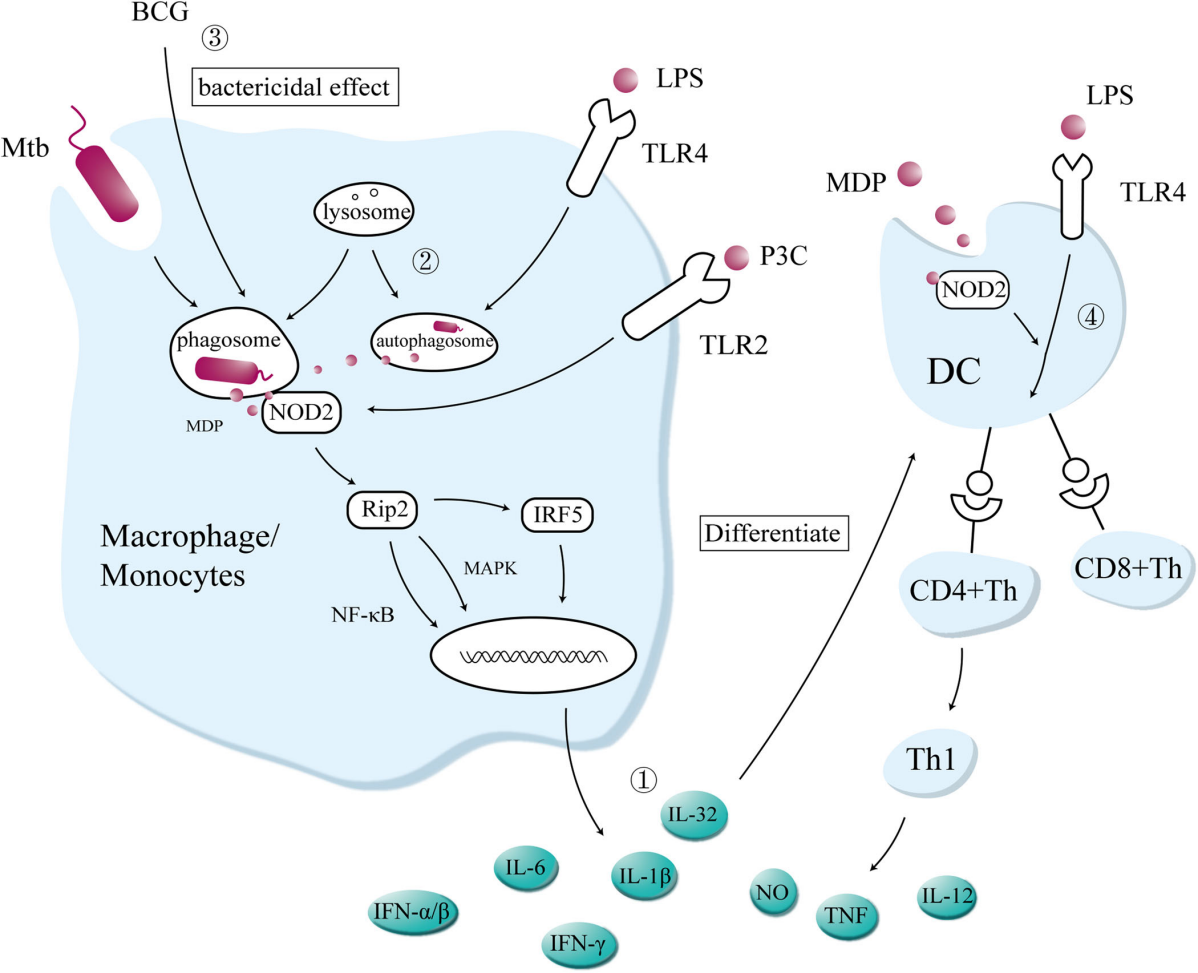
Functions of NOD2 during mycobacterial infection. (1) Macrophages/monocytes phagocytose mycobacteria, which are digested into MDP following the fusion of phagosomes and lysosomes. NOD2 recognizes MDP and produces effectors (TNF, IL‐1β, IL‐6, IL‐12, IL‐32, type I IFNs, and NO) via the RIP2–NF‐κB/MAPK/IRF5 pathway. MDP‐dependent IL‐32 induces the rapid differentiation of monocytes into DCs. (2) NOD2‐dependent autophagy reduces the survival of hidden mycobacteria in MSCs. (3) The NOD2 and TLR4 signaling pathways enhance T‐cell initiation in DCs. (4) BCG vaccines enhances cellular bactericidal efficacy through NOD2‐trained immunity.

In summary, NOD2 mediates immune defense against mycobacterial infections through multiple mechanisms, including activating cytokine production, inducing autophagy, and triggering adaptive immunity via DCs (Figure 3). Notably, its synergistic interaction with TLR4 demonstrates promising potential for host‐directed immunotherapeutic strategies.

### 4.3 Trained Immunity

The BCG vaccine is the longest‐used global vaccine and the only approved vaccine against *Mtb*. The role of trained immunity has recently been identified as the mechanism of BCG‐mediated immunity against *Mtb*, which relies on NOD2 [136]. T stimulation of NOD2‐deficient macrophages with BCG did not increase their cytokine production after heterologous stimulation, suggesting that NOD2 is essential for the establishment of trained immunity [87]. A comparison of PBMCs isolated from volunteers before and 3 months after BCG vaccination revealed that the vaccine caused reprogramming of the innate immune cells induced by NOD2, by epigenetic modification (histone H3 trimethylated at lysine 4). When the cells were re‐exposed to mycobacterial or non‐mycobacterial stimuli, IFN‐γ, TNF‐α, and IL‐1β expression was significantly upregulated, and the expression of the activation markers CD11b and TLR4 increased [87, 137]. The mortality rate of children infected with *Mtb* following BCG vaccination at birth is almost three times lower than that of unvaccinated children [138]. Moreover, the trained immunity is independent of adaptive immunity, because reduced *Mtb* loads were consistently observed 1 week to 1 month after the advance vaccination of T‐cell‐deficient mice with BCG, highlighting the role of macrophages in the BCG‐induced killing of *Mtb* [139] (Figure 3).

These studies highlighted NOD2‐mediated trained immunity as one of the critical factors underlying BCG vaccine efficacy, but deeper investigations into the molecular immunology mechanisms are required to better understand and harness the potential of NOD2‐mediated trained immunity for the prevention and treatment of mycobacterial infections.

### 4.4 Autophagy

In addition to activating the innate and adaptive immune responses through the aforementioned classical immune pathways, the NOD2‐associated regulation of autophagy also contributes to the host defenses against mycobacterial infection. During infection, NOD2‐dependent autophagy acts as an antimicrobial mechanism for clearing intracellular pathogens. The autophagy proteins immunity related GTPase M (IRGM), LC3, and ATG16L1 are recruited into *Mtb* vesicles in an RIP2/p38‐dependent manner and prompt the conversion of LC3‐I to LC3‐II, which is as an indication of autophagosome formation [111, 140, 141]. The N_2_T_4_ signaling pathways also trigger the localization of *Mtb* to lysosomes and induce autophagy to reduce the survival of the *Mtb* hidden in the host's mesenchymal stem cells (MSCs) [142].

Collectively, due to the multifaceted roles of NOD2 in immunity, it exerts immune defense against mycobacterial infections through diverse mechanisms, functioning in a manner that is both genetically and immunologically context‐dependent.

## 5 Perspectives

NOD2, a PRR, transcends conventional microbial detection through its multifaceted immunomodulatory capacity. Beyond merely recognizing MDP, it orchestrates a dual regulatory network. On the one hand, it activates the NF‐κB‐MAPK signaling cascade to enhance pathogen elimination through innate trained immunity, the regulation of autophagy, and synergistic cross‐talk with other PRR systems. On the other hand, it maintains immune homeostasis by suppressing excessive inflammation via tolerance mechanisms and the regulation of autophagy. These intricate functional dimensions offer novel insights into the maintenance of immune homeostasis. Paradoxically, this bidirectional regulatory capacity creates therapeutic dilemmas in disease pathogenesis because the context‐dependent actions of NOD2 may either ameliorate or exacerbate pathological outcomes. Despite its recognized potential as a therapeutic target for inflammatory and infectious disorders, critical gaps remain in our understanding of how to precisely calibrate the immunomodulatory effects of NOD2 across diverse genetic backgrounds, microenvironmental contexts, and disease paradigms. Addressing these knowledge gaps is essential for developing precision‐based therapeutic strategies that harness the pleiotropic functions of NOD2 in a context‐specific manner.

## Author Contributions

**Yi Wang:** data curation, formal analysis, investigation, methodology, visualization, writing – original draft, writing – review and editing. **Zihao Mi:** conceptualization, formal analysis, funding acquisition, investigation, writing – original draft, writing – review and editing. **Hong Liu:** conceptualization, funding acquisition, supervision. **Furen Zhang:** conceptualization, funding acquisition, supervision.

## Conflicts of Interest

The authors declare no conflicts of interest.

## Data Availability

The authors have nothing to report.
